# *N*′-(5-Bromo-2-hydroxy­benzyl­idene)-3-hydroxy­benzohydrazide

**DOI:** 10.1107/S1600536808002250

**Published:** 2008-01-25

**Authors:** Yi Nie

**Affiliations:** a Department of Chemistry, Qufu Normal University, Qufu 273165, People’s Republic of China

## Abstract

The asymmetric unit of the title compound, C_14_H_11_BrN_2_O_3_, contains two crystallographically independent mol­ecules with slightly different conformations with respect to the aromatic rings; the dihedral angles between the two benzene rings in the two mol­ecules are 55.0 (7) and 16.3 (7)°. In the crystal structure, mol­ecules are linked through inter­molecular N—H⋯O, O—H⋯O and O—H⋯N hydrogen bonds, forming chains running along the *a* axis.

## Related literature

For related literature, see: Akitsu & Einaga (2006 ▶); Bahner *et al.* (1968 ▶); Butcher *et al.* (2005 ▶); Hodnett & Mooney (1970 ▶); Merchant & Chothia (1970 ▶); Pradeep (2005 ▶); Sigman & Jacobsen (1998 ▶).
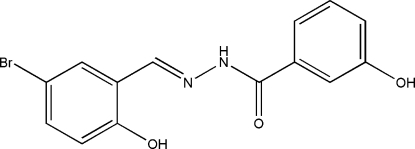


## Experimental

### 

#### Crystal data

C_14_H_11_BrN_2_O_3_
*M* *_r_* = 335.16
Triclinic, 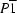

*a* = 6.295 (3) Å
*b* = 14.988 (4) Å
*c* = 15.423 (3) Å
α = 70.97 (2)°
β = 80.64 (2)°
γ = 78.02 (2)°
*V* = 1338.6 (8) Å^3^
*Z* = 4
Mo *K*α radiation
μ = 3.08 mm^−1^
*T* = 298 (2) K
0.20 × 0.18 × 0.18 mm

#### Data collection

Bruker SMART APEX area-detector diffractometer
Absorption correction: multi-scan (*SADABS*; Sheldrick, 1996 ▶) *T*_min_ = 0.549, *T*_max_ = 0.577
11037 measured reflections
5652 independent reflections
2286 reflections with *I* > 2σ(*I*)
*R*_int_ = 0.078

#### Refinement

*R*[*F* ^2^ > 2σ(*F* ^2^)] = 0.072
*wR*(*F* ^2^) = 0.193
*S* = 0.93
5652 reflections
371 parameters
2 restraints
H atoms treated by a mixture of independent and constrained refinement
Δρ_max_ = 0.70 e Å^−3^
Δρ_min_ = −0.50 e Å^−3^

### 

Data collection: *SMART* (Siemens, 1996 ▶); cell refinement: *SAINT* (Siemens, 1996 ▶); data reduction: *SAINT*; program(s) used to solve structure: *SHELXS97* (Sheldrick, 2008 ▶); program(s) used to refine structure: *SHELXL97* (Sheldrick, 2008 ▶); molecular graphics: *SHELXTL* (Sheldrick, 2008 ▶); software used to prepare material for publication: *SHELXL97*.

## Supplementary Material

Crystal structure: contains datablocks global, I. DOI: 10.1107/S1600536808002250/rz2193sup1.cif

Structure factors: contains datablocks I. DOI: 10.1107/S1600536808002250/rz2193Isup2.hkl

Additional supplementary materials:  crystallographic information; 3D view; checkCIF report

## Figures and Tables

**Table 1:** Hydrogen-bond geometry (Å, °)

*D*—H⋯*A*	*D*—H	H⋯*A*	*D*⋯*A*	*D*—H⋯*A*
N4—H4*B*⋯O6^i^	0.90 (5)	2.60 (8)	3.045 (9)	111 (6)
N2—H2⋯O3^ii^	0.90 (6)	2.39 (7)	3.021 (9)	127 (7)
O6—H6⋯O5^iii^	0.82	2.14	2.760 (8)	132
O4—H4⋯N3	0.82	1.95	2.665 (8)	145
O3—H3⋯O2^iv^	0.82	1.93	2.737 (8)	167
O1—H1⋯N1	0.82	1.94	2.654 (8)	145

Symmetry codes: (i) 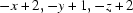; (ii) 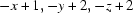; (iii) 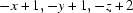; (iv) 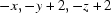.

**Fig. 1.**
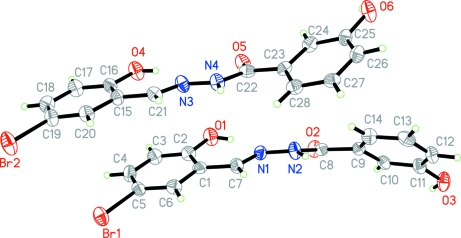
The molecular structure of the title compound with 30% probability ellipsoids.

**Fig. 2.**
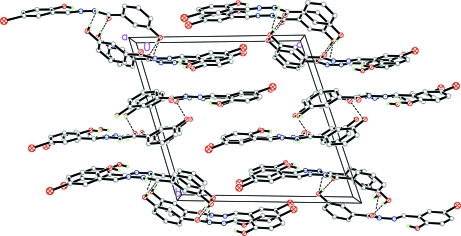
Molecular packing of the title compound. Hydrogen atoms not involved in intermolecular hydrogen bonds (dashed lines) are omitted for clarity.

## supplementary crystallographic information

### Comment

Schiff base compounds have been widely investigated due to their easy synthesis, versatile structures and widely applications (Sigman & Jacobsen, 1998; Akitsu & Einaga, 2006; Pradeep, 2005; Butcher *et al.*, 2005). The excellent antibacterial and antitumor properties of such compounds have attracted much interest in recent years (Hodnett & Mooney, 1970; Bahner *et al.*, 1968; Merchant & Chothia, 1970). In order to further investigate the structures of such compounds, a new Schiff base compound is reported in this paper.

The asymmetric unit of the title compound contains two crystallographically independent molecules (Fig. 1) with slightly different conformation with respect to the aromatic ring planes. The dihedral angles between the two benzene rings in the molecules are 55.0 (7) and 16.3 (7)°, respectively. The molecular conformation is stabilized by intramolecular N—H···O hydrogen bonding interactions (Table 1). In the crystal structure, molecules are linked through intermolecular N–H···O and O–H···O hydrogen bonds (Table 1), forming chains running along the *a* axis (Fig. 2).

### Experimental

The title compound was obtained by stirring of 5-bromosalicylaldehyde (0.1 mmol, 20.1 mg) and 3-hydroxybenzoic acid hydrazide (0.1 mmol, 15.2 mg) in a methanol solution (10 ml) at room temperature. Yellow block-shaped single crystals suitable for X-ray diffraction were formed from the solution after three days.

### Refinement

H2 and H4B were located from a difference Fourier map and refined isotropically, with N–H distances restrained to 0.90 (1) Å, and with *U*_iso_(H) set to 0.08 Å^2^. Other H atoms were positioned geometrically (C–H = 0.93Å and O–H = 0.82 Å) and refined as riding, with *U*_iso_(H) = 1.2*U*_eq_(C) and 1.5*U*_eq_(O).

### Crystal data

**Table:**

C_14_H_11_BrN_2_O_3_	*Z* = 4
*M**_r_* = 335.16	*F*_000_ = 672
Triclinic, *P*1	*D*_x_ = 1.663 Mg m^−^^3^
Hall symbol: -P 1	Mo *K*α radiation λ = 0.71073 Å
*a* = 6.295 (3) Å	Cell parameters from 819 reflections
*b* = 14.988 (4) Å	θ = 2.3–24.3º
*c* = 15.423 (3) Å	µ = 3.08 mm^−^^1^
α = 70.97 (2)º	*T* = 298 (2) K
β = 80.64 (2)º	Block, yellow
γ = 78.02 (2)º	0.20 × 0.18 × 0.18 mm
*V* = 1338.6 (8) Å^3^	

### Data collection

**Table:**

Bruker SMART APEX area-detector diffractometer	5652 independent reflections
Radiation source: fine-focus sealed tube	2286 reflections with *I* > 2σ(*I*)
Monochromator: graphite	*R*_int_ = 0.078
*T* = 298(2) K	θ_max_ = 27.0º
ω scans	θ_min_ = 1.4º
Absorption correction: multi-scan(SADABS; Sheldrick, 1996)	*h* = −7→7
*T*_min_ = 0.549, *T*_max_ = 0.577	*k* = −19→19
11037 measured reflections	*l* = −19→19

### Refinement

**Table:**

Refinement on *F*^2^	Secondary atom site location: difference Fourier map
Least-squares matrix: full	Hydrogen site location: inferred from neighbouring sites
*R*[*F*^2^ > 2σ(*F*^2^)] = 0.072	H atoms treated by a mixture of independent and constrained refinement
*wR*(*F*^2^) = 0.193	*w* = 1/[σ^2^(*F*_o_^2^) + (0.0728*P*)^2^] where *P* = (*F*_o_^2^ + 2*F*_c_^2^)/3
*S* = 0.93	(Δ/σ)_max_ < 0.001
5652 reflections	Δρ_max_ = 0.70 e Å^−^^3^
371 parameters	Δρ_min_ = −0.50 e Å^−^^3^
2 restraints	Extinction correction: none
Primary atom site location: structure-invariant direct methods	

### Special details

**Table:**

Geometry. All e.s.d.'s (except the e.s.d. in the dihedral angle between two l.s. planes) are estimated using the full covariance matrix. The cell e.s.d.'s are taken into account individually in the estimation of e.s.d.'s in distances, angles and torsion angles; correlations between e.s.d.'s in cell parameters are only used when they are defined by crystal symmetry. An approximate (isotropic) treatment of cell e.s.d.'s is used for estimating e.s.d.'s involving l.s. planes.
Refinement. Refinement of *F*^2^ against ALL reflections. The weighted *R*-factor *wR* and goodness of fit *S* are based on *F*^2^, conventional *R*-factors *R* are based on *F*, with *F* set to zero for negative *F*^2^. The threshold expression of *F*^2^ > σ(*F*^2^) is used only for calculating *R*-factors(gt) *etc*. and is not relevant to the choice of reflections for refinement. *R*-factors based on *F*^2^ are statistically about twice as large as those based on *F*, and *R*- factors based on ALL data will be even larger.

### Fractional atomic coordinates and isotropic or equivalent isotropic displacement parameters (Å^2^)

**Table:**

	*x*	*y*	*z*	*U*_iso_*/*U*_eq_	
Br1	0.88585 (14)	0.92277 (7)	0.36574 (6)	0.0631 (3)	
Br2	1.22791 (15)	0.68828 (8)	0.26774 (6)	0.0759 (4)	
O1	0.1995 (9)	0.8047 (5)	0.6860 (4)	0.0657 (17)	
H1	0.2224	0.8173	0.7312	0.099*	
O2	0.1318 (8)	0.8815 (4)	0.9236 (4)	0.0558 (15)	
O3	0.1855 (9)	1.0107 (5)	1.1872 (4)	0.0620 (16)	
H3	0.0778	1.0365	1.1593	0.093*	
O4	0.5513 (9)	0.5809 (5)	0.5941 (4)	0.0649 (17)	
H4	0.5871	0.5866	0.6406	0.097*	
O5	0.5448 (9)	0.6020 (4)	0.8512 (3)	0.0600 (16)	
O6	0.7038 (9)	0.5109 (5)	1.1850 (3)	0.0656 (17)	
H6	0.5797	0.5050	1.1805	0.098*	
N1	0.4379 (10)	0.8497 (4)	0.7878 (4)	0.0450 (16)	
N2	0.4909 (11)	0.8521 (5)	0.8700 (4)	0.0530 (18)	
N3	0.8090 (10)	0.6223 (5)	0.6916 (4)	0.0504 (18)	
N4	0.8723 (10)	0.6282 (5)	0.7716 (4)	0.0491 (17)	
C1	0.5485 (13)	0.8594 (5)	0.6307 (5)	0.047 (2)	
C2	0.3583 (13)	0.8316 (6)	0.6156 (6)	0.050 (2)	
C3	0.3325 (14)	0.8293 (6)	0.5294 (6)	0.058 (2)	
H3A	0.2083	0.8102	0.5207	0.069*	
C4	0.4872 (14)	0.8549 (6)	0.4561 (6)	0.059 (2)	
H4A	0.4682	0.8530	0.3982	0.070*	
C5	0.6726 (13)	0.8837 (5)	0.4693 (5)	0.049 (2)	
C6	0.7046 (12)	0.8877 (5)	0.5549 (5)	0.049 (2)	
H6A	0.8276	0.9088	0.5621	0.059*	
C7	0.5847 (14)	0.8639 (5)	0.7195 (5)	0.051 (2)	
H7	0.7172	0.8774	0.7269	0.061*	
C8	0.3276 (13)	0.8706 (5)	0.9354 (5)	0.044 (2)	
C9	0.3987 (13)	0.8760 (5)	1.0207 (5)	0.044 (2)	
C10	0.2543 (12)	0.9334 (5)	1.0679 (5)	0.045 (2)	
H10	0.1160	0.9607	1.0493	0.054*	
C11	0.3203 (13)	0.9490 (6)	1.1430 (5)	0.052 (2)	
C12	0.5208 (13)	0.9068 (6)	1.1729 (5)	0.053 (2)	
H12	0.5649	0.9182	1.2225	0.064*	
C13	0.6573 (14)	0.8467 (6)	1.1277 (6)	0.061 (2)	
H13	0.7898	0.8149	1.1503	0.073*	
C14	0.6038 (13)	0.8326 (6)	1.0512 (6)	0.056 (2)	
H14	0.7017	0.7950	1.0201	0.067*	
C15	0.9095 (12)	0.6284 (5)	0.5347 (5)	0.044 (2)	
C16	0.7120 (13)	0.6035 (5)	0.5224 (5)	0.046 (2)	
C17	0.6807 (13)	0.5990 (6)	0.4375 (6)	0.057 (2)	
H17	0.5559	0.5784	0.4314	0.069*	
C18	0.8289 (13)	0.6240 (6)	0.3624 (6)	0.056 (2)	
H18	0.8024	0.6229	0.3051	0.067*	
C19	1.0199 (13)	0.6510 (6)	0.3722 (5)	0.052 (2)	
C20	1.0584 (12)	0.6519 (6)	0.4565 (5)	0.049 (2)	
H20	1.1885	0.6687	0.4621	0.059*	
C21	0.9532 (13)	0.6323 (5)	0.6225 (5)	0.048 (2)	
H21	1.0901	0.6425	0.6286	0.058*	
C22	0.7317 (13)	0.6159 (5)	0.8508 (5)	0.046 (2)	
C23	0.8148 (11)	0.6196 (5)	0.9341 (5)	0.0408 (19)	
C24	0.7138 (12)	0.5723 (5)	1.0196 (5)	0.048 (2)	
H24	0.5921	0.5451	1.0220	0.057*	
C25	0.7917 (13)	0.5653 (6)	1.1006 (5)	0.047 (2)	
C26	0.9760 (13)	0.6038 (6)	1.0988 (6)	0.052 (2)	
H26	1.0320	0.5964	1.1534	0.063*	
C27	1.0751 (12)	0.6534 (5)	1.0148 (5)	0.047 (2)	
H27	1.1933	0.6824	1.0133	0.056*	
C28	0.9992 (12)	0.6601 (5)	0.9322 (6)	0.050 (2)	
H28	1.0703	0.6912	0.8761	0.060*	
H2	0.615 (7)	0.860 (6)	0.886 (5)	0.080*	
H4B	1.004 (6)	0.643 (6)	0.771 (5)	0.080*	

### Atomic displacement parameters (Å^2^)

**Table:**

	*U*^11^	*U*^22^	*U*^33^	*U*^12^	*U*^13^	*U*^23^
Br1	0.0515 (6)	0.0820 (7)	0.0483 (6)	−0.0067 (5)	0.0040 (4)	−0.0167 (5)
Br2	0.0608 (6)	0.1244 (10)	0.0477 (6)	−0.0376 (6)	0.0053 (5)	−0.0243 (6)
O1	0.055 (4)	0.095 (5)	0.056 (4)	−0.035 (3)	0.002 (3)	−0.025 (4)
O2	0.038 (3)	0.077 (4)	0.058 (4)	−0.011 (3)	−0.001 (3)	−0.029 (3)
O3	0.043 (3)	0.100 (5)	0.056 (4)	−0.015 (3)	0.002 (3)	−0.042 (4)
O4	0.054 (4)	0.095 (5)	0.051 (4)	−0.033 (3)	−0.002 (3)	−0.017 (4)
O5	0.045 (4)	0.090 (5)	0.048 (3)	−0.031 (3)	−0.005 (3)	−0.013 (3)
O6	0.045 (4)	0.112 (5)	0.042 (3)	−0.028 (4)	−0.001 (3)	−0.019 (3)
N1	0.046 (4)	0.049 (4)	0.042 (4)	−0.005 (3)	0.000 (3)	−0.019 (3)
N2	0.043 (4)	0.076 (5)	0.041 (4)	−0.007 (4)	−0.001 (3)	−0.023 (4)
N3	0.039 (4)	0.067 (5)	0.040 (4)	−0.005 (3)	−0.003 (3)	−0.013 (4)
N4	0.035 (4)	0.071 (5)	0.045 (4)	−0.018 (4)	−0.002 (3)	−0.017 (4)
C1	0.053 (5)	0.034 (5)	0.048 (5)	−0.004 (4)	0.003 (4)	−0.011 (4)
C2	0.041 (5)	0.057 (6)	0.055 (6)	−0.011 (4)	−0.008 (4)	−0.016 (4)
C3	0.052 (6)	0.068 (6)	0.057 (6)	−0.017 (5)	−0.009 (5)	−0.019 (5)
C4	0.061 (6)	0.075 (7)	0.049 (5)	−0.020 (5)	−0.001 (5)	−0.028 (5)
C5	0.051 (5)	0.044 (5)	0.039 (5)	0.008 (4)	0.000 (4)	−0.006 (4)
C6	0.042 (5)	0.052 (6)	0.055 (5)	−0.011 (4)	−0.010 (4)	−0.013 (4)
C7	0.054 (5)	0.058 (6)	0.043 (5)	−0.014 (4)	−0.001 (4)	−0.017 (4)
C8	0.048 (5)	0.040 (5)	0.046 (5)	−0.011 (4)	−0.005 (4)	−0.013 (4)
C9	0.049 (5)	0.041 (5)	0.039 (5)	−0.007 (4)	−0.002 (4)	−0.012 (4)
C10	0.038 (5)	0.053 (5)	0.039 (5)	−0.009 (4)	0.003 (4)	−0.010 (4)
C11	0.046 (5)	0.066 (6)	0.043 (5)	−0.018 (5)	0.001 (4)	−0.013 (4)
C12	0.052 (6)	0.069 (6)	0.040 (5)	−0.010 (5)	−0.009 (4)	−0.016 (4)
C13	0.057 (6)	0.054 (6)	0.058 (6)	0.002 (5)	−0.015 (5)	−0.001 (5)
C14	0.049 (5)	0.062 (6)	0.057 (6)	−0.007 (5)	−0.007 (4)	−0.019 (5)
C15	0.039 (5)	0.057 (6)	0.040 (5)	−0.012 (4)	−0.006 (4)	−0.015 (4)
C16	0.049 (5)	0.050 (5)	0.041 (5)	−0.014 (4)	−0.008 (4)	−0.011 (4)
C17	0.046 (5)	0.076 (7)	0.057 (6)	−0.020 (5)	−0.008 (5)	−0.021 (5)
C18	0.049 (5)	0.065 (6)	0.059 (6)	−0.008 (5)	−0.016 (5)	−0.023 (5)
C19	0.047 (5)	0.067 (6)	0.041 (5)	−0.010 (4)	−0.005 (4)	−0.017 (4)
C20	0.037 (5)	0.064 (6)	0.052 (5)	−0.022 (4)	−0.001 (4)	−0.018 (4)
C21	0.041 (5)	0.053 (6)	0.047 (5)	−0.009 (4)	−0.007 (4)	−0.009 (4)
C22	0.036 (5)	0.041 (5)	0.056 (5)	−0.012 (4)	0.000 (4)	−0.009 (4)
C23	0.031 (4)	0.041 (5)	0.056 (5)	−0.005 (4)	−0.012 (4)	−0.019 (4)
C24	0.033 (4)	0.057 (6)	0.059 (5)	−0.023 (4)	0.011 (4)	−0.024 (4)
C25	0.040 (5)	0.058 (6)	0.049 (5)	−0.002 (4)	−0.005 (4)	−0.025 (4)
C26	0.045 (5)	0.061 (6)	0.056 (5)	−0.007 (4)	−0.006 (4)	−0.027 (5)
C27	0.042 (5)	0.040 (5)	0.061 (5)	−0.013 (4)	−0.012 (4)	−0.012 (4)
C28	0.044 (5)	0.049 (5)	0.059 (5)	−0.015 (4)	0.001 (4)	−0.015 (4)

### Geometric parameters (Å, °)

**Table:**

Br1—C5	1.928 (7)	C9—C14	1.400 (10)
Br2—C19	1.912 (8)	C9—C10	1.401 (9)
O1—C2	1.368 (9)	C10—C11	1.395 (10)
O1—H1	0.8200	C10—H10	0.9300
O2—C8	1.245 (9)	C11—C12	1.369 (11)
O3—C11	1.392 (9)	C12—C13	1.388 (10)
O3—H3	0.8200	C12—H12	0.9300
O4—C16	1.377 (8)	C13—C14	1.369 (11)
O4—H4	0.8200	C13—H13	0.9300
O5—C22	1.235 (8)	C14—H14	0.9300
O6—C25	1.388 (9)	C15—C20	1.396 (9)
O6—H6	0.8200	C15—C16	1.427 (10)
N1—C7	1.276 (8)	C15—C21	1.447 (10)
N1—N2	1.375 (8)	C16—C17	1.380 (10)
N2—C8	1.371 (9)	C17—C18	1.365 (10)
N2—H2	0.90 (6)	C17—H17	0.9300
N3—C21	1.273 (8)	C18—C19	1.391 (11)
N3—N4	1.390 (8)	C18—H18	0.9300
N4—C22	1.371 (9)	C19—C20	1.365 (10)
N4—H4B	0.90 (5)	C20—H20	0.9300
C1—C6	1.410 (10)	C21—H21	0.9300
C1—C2	1.422 (10)	C22—C23	1.485 (10)
C1—C7	1.450 (10)	C23—C24	1.397 (10)
C2—C3	1.377 (10)	C23—C28	1.410 (10)
C3—C4	1.374 (10)	C24—C25	1.380 (10)
C3—H3A	0.9300	C24—H24	0.9300
C4—C5	1.391 (11)	C25—C26	1.392 (10)
C4—H4A	0.9300	C26—C27	1.386 (10)
C5—C6	1.388 (10)	C26—H26	0.9300
C6—H6A	0.9300	C27—C28	1.398 (10)
C7—H7	0.9300	C27—H27	0.9300
C8—C9	1.488 (10)	C28—H28	0.9300
			
C2—O1—H1	109.5	C14—C13—C12	122.3 (8)
C11—O3—H3	109.5	C14—C13—H13	118.8
C16—O4—H4	109.5	C12—C13—H13	118.8
C25—O6—H6	109.5	C13—C14—C9	118.7 (8)
C7—N1—N2	116.6 (7)	C13—C14—H14	120.7
C8—N2—N1	119.5 (6)	C9—C14—H14	120.7
C8—N2—H2	107 (5)	C20—C15—C16	116.6 (7)
N1—N2—H2	132 (5)	C20—C15—C21	120.9 (7)
C21—N3—N4	115.7 (7)	C16—C15—C21	122.5 (7)
C22—N4—N3	120.6 (6)	O4—C16—C17	118.2 (7)
C22—N4—H4B	119 (5)	O4—C16—C15	121.7 (7)
N3—N4—H4B	120 (5)	C17—C16—C15	120.1 (7)
C6—C1—C2	118.4 (7)	C18—C17—C16	121.5 (8)
C6—C1—C7	118.5 (8)	C18—C17—H17	119.3
C2—C1—C7	123.1 (7)	C16—C17—H17	119.3
O1—C2—C3	118.5 (7)	C17—C18—C19	119.3 (8)
O1—C2—C1	121.2 (7)	C17—C18—H18	120.3
C3—C2—C1	120.4 (8)	C19—C18—H18	120.3
C4—C3—C2	121.1 (8)	C20—C19—C18	120.0 (7)
C4—C3—H3A	119.4	C20—C19—Br2	119.8 (6)
C2—C3—H3A	119.4	C18—C19—Br2	120.2 (6)
C3—C4—C5	119.3 (8)	C19—C20—C15	122.4 (7)
C3—C4—H4A	120.4	C19—C20—H20	118.8
C5—C4—H4A	120.4	C15—C20—H20	118.8
C6—C5—C4	121.6 (7)	N3—C21—C15	121.7 (7)
C6—C5—Br1	119.2 (7)	N3—C21—H21	119.2
C4—C5—Br1	119.2 (6)	C15—C21—H21	119.2
C5—C6—C1	119.3 (8)	O5—C22—N4	120.2 (7)
C5—C6—H6A	120.4	O5—C22—C23	122.6 (7)
C1—C6—H6A	120.4	N4—C22—C23	117.2 (7)
N1—C7—C1	121.1 (8)	C24—C23—C28	118.4 (7)
N1—C7—H7	119.5	C24—C23—C22	117.1 (7)
C1—C7—H7	119.5	C28—C23—C22	124.2 (7)
O2—C8—N2	121.4 (7)	C25—C24—C23	121.1 (7)
O2—C8—C9	122.5 (7)	C25—C24—H24	119.5
N2—C8—C9	116.1 (7)	C23—C24—H24	119.5
C14—C9—C10	119.9 (7)	C24—C25—O6	120.9 (7)
C14—C9—C8	123.5 (7)	C24—C25—C26	120.6 (8)
C10—C9—C8	116.5 (7)	O6—C25—C26	118.2 (7)
C11—C10—C9	119.3 (7)	C27—C26—C25	119.3 (8)
C11—C10—H10	120.4	C27—C26—H26	120.4
C9—C10—H10	120.4	C25—C26—H26	120.4
C12—C11—O3	118.7 (7)	C26—C27—C28	120.6 (7)
C12—C11—C10	120.9 (8)	C26—C27—H27	119.7
O3—C11—C10	120.3 (7)	C28—C27—H27	119.7
C11—C12—C13	118.8 (8)	C27—C28—C23	119.9 (7)
C11—C12—H12	120.6	C27—C28—H28	120.0
C13—C12—H12	120.6	C23—C28—H28	120.0

### Hydrogen-bond geometry (Å, °)

**Table:**

*D*—H···*A*	*D*—H	H···*A*	*D*···*A*	*D*—H···*A*
N4—H4B···O6^i^	0.90 (5)	2.60 (8)	3.045 (9)	111 (6)
N2—H2···O3^ii^	0.90 (6)	2.39 (7)	3.021 (9)	127 (7)
O6—H6···O5^iii^	0.82	2.14	2.760 (8)	132
O4—H4···N3	0.82	1.95	2.665 (8)	145
O3—H3···O2^iv^	0.82	1.93	2.737 (8)	167
O1—H1···N1	0.82	1.94	2.654 (8)	145

Symmetry codes: (i) −*x*+2, −*y*+1, −*z*+2; (ii) −*x*+1, −*y*+2, −*z*+2; (iii) −*x*+1, −*y*+1, −*z*+2; (iv) −*x*, −*y*+2, −*z*+2.
